# Protective Activities of *Dendrobium huoshanense* C. Z. Tang et S. J. Cheng Polysaccharide against High-Cholesterol Diet-Induced Atherosclerosis in Zebrafish

**DOI:** 10.1155/2020/8365056

**Published:** 2020-07-08

**Authors:** Xiangcheng Fan, Jichun Han, Lijun Zhu, Zhipeng Chen, Jiajing Li, Yue Gu, Feng Wang, Tao Wang, Yunyun Yue, Jing Shang

**Affiliations:** ^1^School of Traditional Chinese Pharmacy, China Pharmaceutical University, Nanjing, 211198 Jiangsu, China; ^2^Nanjing Drum Tower Hospital Clinical College of Nanjing Medical University, Nanjing, 211166 Jiangsu, China; ^3^Department of Cardiology, Nanjing First Hospital, Nanjing Medical University, Nanjing, 210029 Jiangsu, China; ^4^Jiangsu Center for Pharmacodynamics Research and Evaluation, China Pharmaceutical University, Nanjing 210009, China

## Abstract

Cardiovascular disease is the highest cause of death, and atherosclerosis (AS) is the primary pathogenesis of many cardiovascular diseases. In this study, we aim to investigate the possible pharmaceutical effects of *Dendrobium huoshanense* C. Z. Tang et S. J. Cheng polysaccharide (DHP) in AS. We fed zebrafish with high-cholesterol diet (HCD) to establish a zebrafish AS model and treated with DHP and observed plaque formation and neutrophil counts under a fluorescence microscope. Next, a parallel flow chamber was utilized to establish low shear stress- (LSS-) induced endothelial cell (EC) dysfunction model. We observed that DHP significantly improved HCD-induced lipid deposition, oxidative stress, and inflammatory response, mainly showing that DHP significantly increased superoxide dismutase (SOD) activity, decreased plaque formation, and decreased neutrophil recruitment and the levels of total cholesterol (TC), triglyceride (TG), malondialdehyde (MDA), and reactive oxygen species (ROS). Furthermore, DHP significantly improved LSS-induced oxidative stress and EC dysfunction. Our results indicated that DHP can exert treatment effects on AS, which may attribute to its hypolipidemic, antioxidant, anti-inflammatory activities and improving LSS-induced EC dysfunction. DHP has promising potential for further development as a functional natural medicine source targeted at AS prevention.

## 1. Introduction

Due to the improvement of living conditions, diseases caused by high-cholesterol diet (HCD) become more and more obvious. HCD can affect the body's various indicators that are related to many diseases, such as obesity and atherosclerosis (AS) [1]. AS is known as the deposition of blood components such as lipids in the intima of the arteries, the proliferation of smooth muscle cells, the increase of collagen fibers, and formatting porridge-like lipid-containing necrotic lesions and vascular wall sclerosis [2, 3]. Recently, with the gradual increase of incidence, AS is also becoming the main cause of coronary heart disease, cerebral infarction, and peripheral vascular disease, which seriously threatens people's health [4, 5]. Thus, there is a pressing need for an economical and effective method to prevent the progression of AS.

The pathogenesis of AS is complex. Hypercholesterolemia is an important risk factor for the occurrence and development of AS, and it is also the pathophysiological basis of cardiovascular diseases [6]. HCD raises blood lipid levels, causing lipids to accumulate in blood vessels, forming early AS plaques. HCD also increases reactive oxygen species (ROS) activity and malondialdehyde (MDA) content, reduces some antioxidant enzyme activities (e.g., superoxide dismutase [SOD]), breaks the redox equilibrium, and promotes AS. AS is a chronic inflammation and autoimmune disease; there is a variety of immune cells involved in the occurrence of AS, such as macrophages, lymphocytes, dendritic cells, and neutrophils. In early AS, neutrophils are abundantly recruited in blood vessels by activating macrophages and accelerating foam cell formation, which promoted plaque instability [7]. Shear stress is also involved in the development of AS. Shear stress, a force between blood flow and blood vessel endothelium for the unit area of the blood vessel wall, was mainly divided into low shear stress (LSS), high shear stress, laminar shear stress, and oscillations shear stress [8]. LSS can induce endothelial cell (EC) dysfunction, such as oxidative stress and EC proliferation, eventually leading to plaque formation. LSS also promotes AS by priming EC for enhanced expression of inflammatory molecules (e.g., intercellular adhesion molecule-1 [ICAM-1] and vascular cell adhesion molecule-1 [VCAM-1]) [9].

Currently, the drugs used to treat AS are mainly lipid-lowering drugs. However, since the pathogenesis of AS is complex, long-term and high-dose applications of single drug therapies, like simvastatin which targets single molecule, can produce some side effects, such as myopathy and liver damage [10]. Therefore, finding a drug to prevent AS may be a safe and effective strategy.

*Dendrobium*, as one of the largest genera in the *Orchidaceae*, has more than 1500 species [11]. *Dendrobium huoshanense* C. Z. Tang et S. J. Cheng (DH) is one of the *Dendrobium*, which is only distributed in China. More recently, DH has garnered more attention due to its excellent and extensive bioactivity merits, such as improving human immunity, antidiabetes, hepatoprotective, anti-angiogenesis, anti-inflammatory, and anticancer benefits [12]. Emerging evidence has identified the bioactive ingredients of DH, such as polysaccharides, alkaloids, and flavonoids [13, 14]. It is worthy that the polysaccharides as the main active compounds have exerted numerous functions including alleviating lung inflammation [15], inhibiting epithelial cell apoptosis [16], antioxidant [17], and regulating hepatic glucose homeostasis [18]. However, it is not clear now whether *Dendrobium huoshanense* C. Z. Tang et S. J. Cheng polysaccharide (DHP) has effects on AS, and the mechanism involved in the progression of AS remains to be fully elucidated.

In this study, we fed zebrafish with HCD to establish a zebrafish AS model and explored the effects of DHP on plaque formation, lipid levels, inflammation, and oxidative stress. We also use a parallel flow chamber to establish LSS-induced EC dysfunction model and observed the effects of DHP on LSS-induced oxidative stress and EC dysfunction in EA.hy 926 cells.

## 2. Materials and Methods

### 2.1. Reagents

DH was purchased from Anhui Yuanhe Chinese Medicine Development Co., Ltd. (Hefei, China). SOD was provided from Wuhan Elabscience Biotechnology Co., Ltd (Wuhan, China). Dihydroethidium (DHE), 2, 7-Dichlorodi-hydrofluorescein diacetate (DCFH-DA), and 4-Amino-5-aminomethyl-2′,7′-difluorescein diacetate (DAF-FM DA) were purchased from Nanjing Beyotime Biotechnology Co., Ltd (Nanjing, China). MTT was purchased from Sigma-Aldrich (St. Louis, MO, United States). All of the other reagents were of analytical grade.

### 2.2. Preparation of DHP

The extraction and purification of DHP were executed as described previously [19]. Briefly, the dried protocorm-like body was ground into a powder, then weighted 250 g powder in distilled water and heated at 80°C for 3 h; the ratio of material to liquid is 1 : 3 (g : mL). The extracts were combined with the powder after being extracted three times and concentrated to 1 L by a rotary evaporator. The protein was precipitated with ethanol for 4 h. After deproteinization with the Sevag method [20] in the crude polysaccharide, the polysaccharide was redissolved in distilled water again and subjected to DEAE-cellulose column (1.6 × 60 cm), followed by a gradient elution with distilled water and NaCl (0.1, 0.2, 0.3, and 0.6 M) at a flow rate of 5.0 mL/min. The impurities were removed by centrifugation at 8000 g.

### 2.3. Zebrafish AS Model

**Figure 1:**
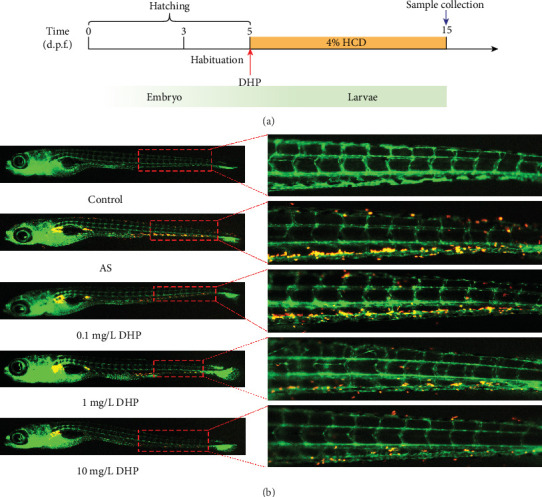
DHP reduced the formation of plaques in AS zebrafish. (a) Schematic representation of the experimental procedure. (b) Hypercholesterolemic zebrafish larvae induced by 4% red fluorescence-labeled HCD for 10 days. Red dotted square denoted the main location of lipid accumulation.

Lipid accumulation in zebrafish blood vessels was detected to reflect early atherosclerotic plaque formation. Five-day old Tg (*fli1: EGFP*) (endothelial EGFP) zebrafish larvae were fed with 4% cholesterol (supplemented with 10 *μ*g/g of red fluorescent lipid) diet treated with or without DHP (0.1, 1, and 10 mg/L) for 10 days (Figure 1(a)) [21]. Zebrafish larvae were fed only with 10 *μ*g/g of red fluorescent lipid (not contain 4% cholesterol) for 10 days as control. A large amount of red fluorescent lipid accumulation in the zebrafish green blood vessels can be observed under a fluorescence microscope. These accumulated lipids are similar to the plaques of early AS [22]. All animal experiments were permitted by Jiangsu Provincial standard ethical guidelines for the use of experimental animals under the ethical committees mentioned above (SYXK(SU)2016-0011, 27 January 2016).

### 2.4. Detection of Inflammation in AS Zebrafish

Studies have found that Tg (*mpx:EGFP*) zebrafish (neutrophils are specifically labeled with green fluorescent protein) can be used to observe inflammatory responses. According to the references [23], we observe inflammation in Tg (*mpx: EGFP*) zebrafish. After 10 days of feeding and treatment and fasting for 24 h, the number of central granulocytes was observed under a fluorescence microscope to reflect inflammation in zebrafish.

### 2.5. Nile Red Staining and Biochemical Measurement

The five-day old wild-type AB-line zebrafish larvae were randomly divided into five groups. After 10 days of feeding, fasting for 24 h, and using Nile Red staining to detect lipid levels in each group of zebrafish. The zebrafish larvae were collected and rinsed with PBS. After that, the zebrafish were prepared with 1 *μ*g/mL Nile Red working fluid and mixed in the dark for 30 min, then washed twice with egg water. DCFH-DA was used to detect ROS expression in each group of zebrafish. All zebrafish were treated at the same time after the cleaning was completed; the zebrafish larvae were anesthetized with 0.05% Tricaine. Body steatosis was observed under a stereomicroscope (Olympus SZX 16) and photographed. Simultaneously, forty larvae in each group were randomly selected and sacrificed as one sample, and three samples were prepared for testing each index. TG, TC levels, SOD activity, and MDA levels (Jiancheng, Nanjing, China) were measured following the manufacturer's instructions.

### 2.6. Cell Culture and Treatments

The human umbilical vein endothelial cell (HUVEC) line EA.hy 926 cells were cultured in RPMI 1640 (Invitrogen) medium supplemented with 10% (*v*/*v*) fetal bovine serum (FBS), 100 U/mL penicillin, and 100 *μ*g/mL streptomycin (Gibco, Grand Island, NY) and cultured at 37°C in a ventilated with 95% humidified atmosphere incubator containing 5% CO_2_. When EA.hy 926 cells reached a confluence of 60-70%, they were passaged with 0.25% trypsin (*w*) and plated on a glass slide (30 × 50 mm, 1 × 10^5^ cells/mL). And these cells were pretreated with DMSO (0.1%) or different concentration of DHP (0.1, 1, and 10 mg/L) in serum-free RPMI 1640, after cells treated with or without DHP for 24 h, then followed by LSS experiment for the indicated time.

### 2.7. Low Shear Stress

The parallel-plate flow chamber (Shanghai Medical Instrument School, Shanghai, China) was performed to impose an LSS on the monolayer of EA.hy 926 cells as previously described by Zhang et al. [24]; the laminar with tiled EA.hy 926 cells were placed on the chamber and obtained an LSS (3 dyn/cm^2^) in the incubator at 37°C for 30 min. The cells under static without flow were used as control.

### 2.8. Assay of EC Dysfunction and Oxidative Stress

Cell culture medium was collected to measure secreted endothelin-1 (ET-1) level, nitric oxide (NO) level, and prostaglandin I2 (PGI_2_) level using an ET-1, NO, and PGI_2_ ELISA kit (Shanghai Enzyme Biotechnology Co., Ltd., Shanghai, China). EA.hy 926 cells were collected to measure the secretion of SOD activity, MDA, glutathione (GSH), and glutathione disulfide (GSSG) content using the commercial kits (Beijing Solarbio Science & Technology Co., Ltd., Beijing, China) and following manufacturer's instructions.

### 2.9. Quantitative Real-Time PCR

Total RNA of the cells was extracted using the Trizol Reagent (Invitrogen, Carlsbad, CA, USA), according to the manufacturer's instructions. The RNA was reverse-transcribed using the PrimeScript RT Master Mix Perfect Real Time and following the manufacturer's protocol. The resultant cDNA was applied as template for quantitative PCR analyses in the Thermal Cycler Dice® Real Time System (Takara Bio Inc., Shiga, Japan) with the following sets of primers; primers for qPCR were designed by Primer3 software and are listed in Supplementary Table 1. The mRNA expression data are expressed as relative expression ratio normalized to GAPDH.

### 2.10. Statistics Analysis

All data were expressed as mean ± standard deviation (SD). Multiple comparisons were made using the one-way analysis of variance (ANOVA) and Student's *t*-test for unpaired samples. All statistical analysis was performed by Graphpad Prism 8.0 software (San Diego, CA, USA). *p* < 0.05 was considered having a significant difference.

## 3. Results

### 3.1. DHP Reduced the Formation of Plaques in AS Zebrafish

We observed a large amount of lipid deposited near of the blood vessel tail of zebrafish in the AS group, but without any changes in the control group (Figure 1(b)). Intriguingly, compared with the AS group, the bath administration of 1 mg/L and 10 mg/L DHP significantly decreased the lipid deposits in zebrafish larvae. However, no significant difference was found between the AS group and 0.1 mg/L DHP group.

### 3.2. DHP Improved Lipid Metabolism Homeostasis and Oxidative Stress in AS Zebrafish

**Figure 2:**
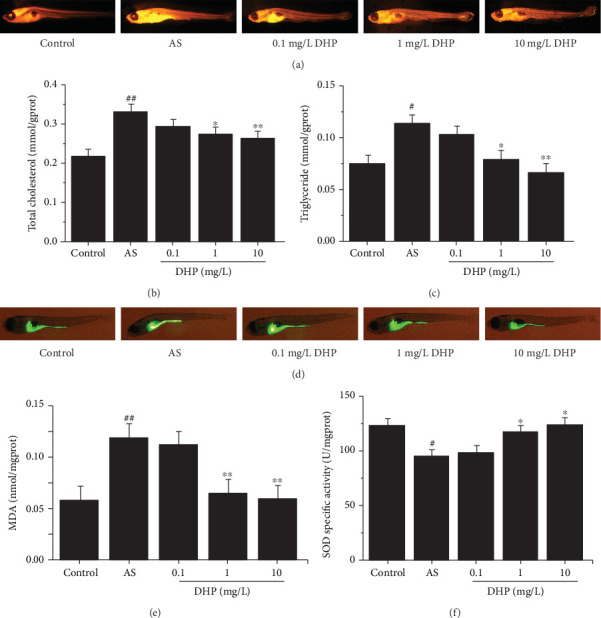
DHP improved lipid metabolism homeostasis and oxidative stress in AS zebrafish. (a) Representative images of Nile Red staining of AS zebrafish larvae treated with 0.1, 1, and 10 mg/L DHP for 10 days. (b) TC and (c) TG contents in whole mount of zebrafish larvae (*n* = 40). (d) Detected the production of ROS by DCFH-DA and captured using stereomicroscope (green fluorescence). Oxidized species content of (e) MDA and (f) SOD was quantified in the whole body of zebrafish larvae (*n* = 40). SD was depicted as vertical bars. ^#^*p* < 0.05, ^##^*p* < 0.01, compared with control group; ^∗^*p* < 0.05, ^∗∗^*p* < 0.01, compared with AS group. Significance was calculated by one-way ANOVA followed by unpaired *t*-test. *n* represents the numbers of zebrafish larvae.

Compared with control group, the lipid level significantly increased in AS group, which was notably reversed by 1 mg/L and 10 mg/L DHP (Figure 2(a)). Consistently, TC and TG level was also significantly decreased in AS zebrafish treated with 1 mg/L and 10 mg/L DHP, but without any change of 0.1 mg/L DHP (Figures 2(b) and 2(c)). In summary, DHP could efficiently down-regulate lipid levels in AS zebrafish.

ROS level significantly increased in AS group compared with control group (Figure 2(d)). But the ROS level was markedly decreased when treated with 1 mg/L and 10 mg/L DHP, and still 0.1 mg/L DHP had no change. With MDA content and SOD activity, it is the same reversion induced by DHP in zebrafish (Figures 2(e) and 2(f)).

### 3.3. DHP Protected against Inflammation in AS Zebrafish

**Figure 3:**
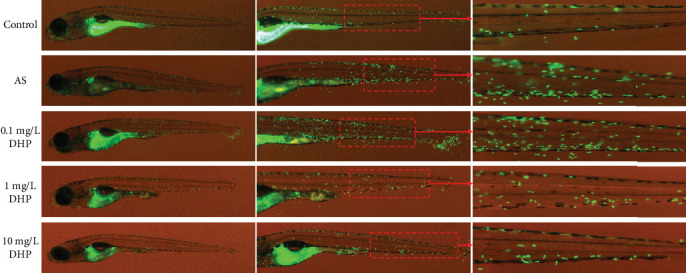
DHP protected against inflammation in AS zebrafish. Anti-inflammatory effect of DHP in 4% HCD induced inflammation model of the Tg (*mpx: EGFP*) zebrafish larvae. Squares mark the specific regions of near the tail of zebrafish larvae (red). The green fluorescence indicates the levels of inflammation emitted by the neutrophils.

It is generally believed that neutrophil is a major cell type that causes tissue damage accompanied by severe inflammation. To identify the characterization of inflammatory response in the process of early states of AS, first, we generated a transgenic zebrafish line that overexpresses green fluorescent protein of neutrophils in transgenic *mpx: EGFP* zebrafish. We found a remarkable increase of green fluorescent-labeled neutrophils at the sites of vascular in AS group zebrafish larvae (Figure 3). Thereafter, in order to test the efficacy of DHP as a therapeutic drug that would restrain green fluorescent-labeled neutrophil migration and inflammatory response, we performed different doses of DHP in zebrafish larvae induced by HCD diet. We observed specific diminishing of neutrophil contents in the tail of zebrafish larvae from both the 1 mg/L and 10 mg/L DHP treatments, but without alteration in the level of 0.1 mg/L. In summary, DHP protected against oxidative stress and inflammation in AS zebrafish.

### 3.4. DHP Improved LSS-Induced EC Dysfunction

**Figure 4:**
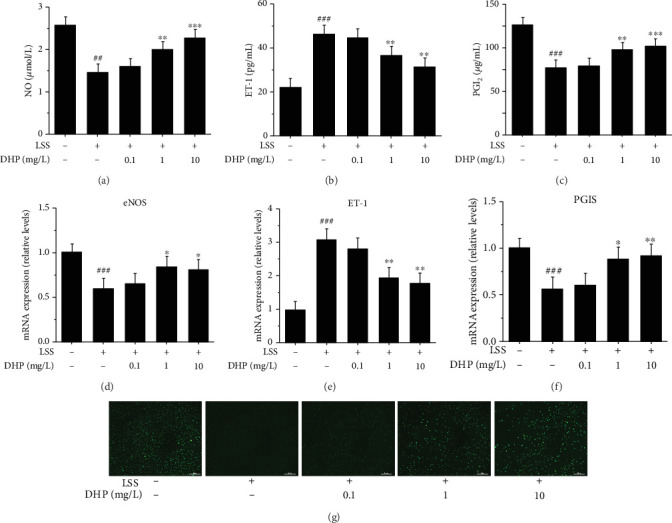
DHP improved LSS-induced EC dysfunction. (a) NO, (b) ET-1, and (c) PGI2 levels after EA.hy 926 cells were exposed to LSS by a parallel flow chamber with DHP (0.1, 1, and 10 mg/L) treatment or not. The mRNA expression of (d) eNOS, (e) ET-1, and (f) PGIS by RT-qPCR (*n* = 3). (g) The fluorescence intensity of NO after EA.hy 926 cells were exposed to LSS by a parallel flow chamber. SD was depicted as vertical bars. ^##^*p* < 0.05, ^###^*p* < 0.001, compared with the control group (LSS, 0 min); ^∗^*p* < 0.05, ^∗∗^*p* < 0.01, ^∗∗∗^*p* < 0.001, compared with the LSS group (LSS, 30 min). Significance was calculated by one-way ANOVA followed by unpaired *t*-test. *n* represents the independent experiments.

EA.hy 926 cells were exposed to laminar flow with a value of 0 or 3 dyn/cm^2^ for 30 min [25]. LSS significantly reduced the release of NO and PGI_2_ and significantly increased the release of ET-1, which was significantly inhibited by 1 mg/L and 10 mg/L DHP, but not by 0.1 mg/L DHP (Figures 4(a)–4(c)). Furthermore, LSS significantly reduced the mRNA levels of endothelia NO synthase (eNOS) and prostaglandin I2 synthase (PGIS) and significantly increased the ET-1 mRNA level, which was significantly inhibited by 1 mg/L and 10 mg/L DHP, but not by 0.1 mg/L DHP (Figures 4(d)–4(f)). We examined intracellular NO activities using the fluorescent probe DAF-FM DA. LSS significantly reduced NO level in EA.hy 926 cell, and 1 mg/L and 10 mg/L DHP significantly increased NO level, but 0.1 mg/L DHP did not improve LSS-induced decrease in NO level (Figure 4(g)). Briefly, DHP have effective improvement on LSS-induced EC dysfunction.

### 3.5. DHP Improved LSS-Induced Oxidative Stress and Inflammation

**Figure 5:**
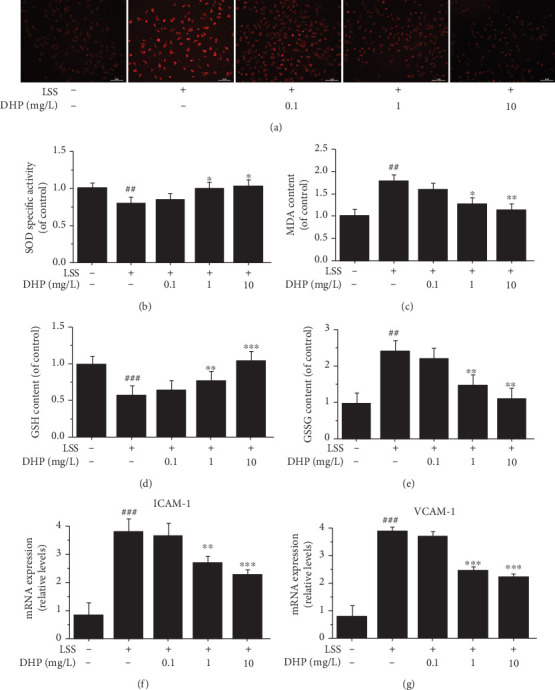
DHP improved LSS-induced oxidative stress and inflammation. (a) ROS levels after EA.hy 926 cells were exposed to LSS by a parallel flow chamber with DHP (0.1, 1, and 10 mg/L) treatment or not. Oxidized species content of (b) SOD, (c) MDA, (d) GSH, and (e) GSSG was quantified in EA.hy 926 cells induced by LSS. The mRNA expression of (f) ICAM-1 and (g) VCAM-1 by RT-qPCR (*n* = 3). SD was depicted as vertical bars. ^##^*p* < 0.01, ^###^*p* < 0.001, compared with the control group (LSS, 0 min); ^∗^*p* < 0.05, ^∗∗^*p* < 0.01, ^∗∗∗^*p* < 0.001, compared with the LSS group (LSS, 30 min). Significance was calculated by one-way ANOVA followed by unpaired *t*-test. *n* represents the independent experiments.

LSS significantly induced ROS level, which was significantly inhibited by 1 mg/L and 10 mg/L DHP, but not by 0.1 mg/L DHP (Figure 5(a)). We also observed that LSS significantly reduced SOD and GSH levels and significantly increased MDA and GSSG levels, which was significantly inhibited by 1 mg/L and 10 mg/L DHP, but not by 0.1 mg/L DHP in EA.hy 926 cells (Figures 5(b)–5(e)).

Later on, we further determined whether LSS affects the expression of adhesion molecules or not. Our data illustrated a dramatical increase of ICAM-1 and VCAM-1 expression after treated with LSS (Figures 5(f) and 5(g)). Moreover, as compared to the LSS group, the changes of these adhesion molecules were downregulated with the doses of 1 and 10 mg/L DHP, respectively. Thus, our findings signified that DHP would alleviate LSS-induced oxidative stress and inflammation in EA.hy 926 cells.

### 3.6. Effect of DHP on LSS-Related Target mRNA Expression

**Figure 6:**
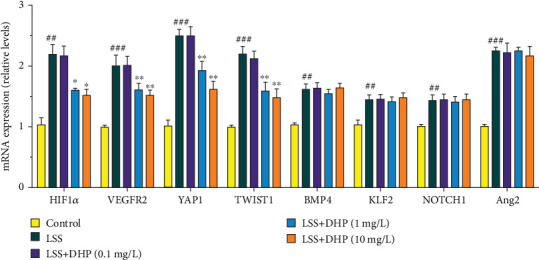
Effect of DHP on LSS-related target mRNA expression. The mRNA expression of HIF1*α*, VEGFR2, YAP1, TWIST1, BMP4, KLF2, NOTCH1, and Ang2 was examined by RT-qPCR (*n* = 3). SD was depicted as vertical bars. ^##^*p* < 0.01, ^###^*p* < 0.001, compared with the control group (LSS, 0 min); ^∗∗^*p* < 0.01, ^∗∗∗^*p* < 0.001, compared with the LSS group (LSS, 30 min). Significance was calculated by one-way ANOVA followed by unpaired *t*-test. *n* represents the independent experiments.

Some mechanosensitive molecules are involved in the process of LSS-induced AS, such as bone morphogenetic protein 4 (BMP4), Krüppel-like factor 2 (KLF2), hypoxia-inducible factor 1*α* (HIF1*α*), vascular endothelial growth factor receptor 2 (VEGFR2), Columba livia notch1 (NOTCH1), yes-associated protein (YAP1), angiopoietin2 (Ang2), and twist-related protein 1 (TWIST1). To investigate the possible role of DHP in improving LSS-induced EC dysfunction, we examined the effect of DHP on mRNA expression of these mechanosensitive molecules. LSS significantly increased the mRNA levels of BMP4, KLF2, HIF1*α*, VEGFR2, NOTCH1, YAP1, Ang2, and TWIST1 (Figure 6). DHP significantly decreased the mRNA levels of HIF1*α*, VEGFR2, YAP1, and TWIST1, but could not decrease the mRNA expression of BMP4, KLF2, NOTCH1, and Ang2. This result suggests that DHP may ameliorate LSS-induced EC disorders by inhibiting mRNA expression of HIF1*α*, VEGFR2, YAP1, and TWIST1.

## 4. Discussion

Lipid metabolism disorder is the basis of AS lesions, which is characterized by the involvement of the affected arterial lesions from the intima, usually with the accumulation of lipids, forming early plaques. In the present study, we fed zebrafish with HCD to establish a zebrafish AS model, we found that a large number of lipids accumulate in the blood vessels of AS zebrafish, and most of these lipid accumulation sites are blood vessels at the LSS, which is consistent with the predisposition of AS plaques (Figure S1) (Figure 1(b)). Intriguingly, DHP significantly improved lipid accumulation in the blood vessels at the LSS of AS zebrafish. These results indicate that DHP can inhibit plaque formation in AS.

It is generally considered that hyperlipidemia, inflammation, and oxidative stress lead to the initiation and development of AS [26–28]. In the present study, zebrafish in the AS model group showed hyperlipidemia, oxidative stress, and inflammation as expected. These results further illustrate that zebrafish can be used to study AS. Interestingly, DHP significantly improved the HCD-induced hyperlipaemia, oxidative stress, and inflammation. These results also indicate that the anti-AS effect of DHP may be related to its lipid-lowering, antioxidant, and anti-inflammatory effects.

Some studies have found that LSS plays an important role in the occurrence of AS [29]. LSS destroys the balance of the release of some vasoactive substances in vascular endothelial cells, causing EC dysfunction, which is the main cause of AS [30, 31]. EC dysfunction is characterized by an imbalance between vasodilatory and vasoconstrictive molecules. These molecules are synthesized and released by EC and include PGI_2_, NO, and ET-1. PGI_2_ and NO are effective vasodilators and are produced by PGIS and eNOS, respectively, while ET-1 is a potent vasoconstrictor [32]. And high levels of ET-1 are considered to be one of the risk factors for AS [33]. In the present study, we used a parallel flow chamber to establish LSS-induced EC dysfunction model. We found that LSS significantly reduced the release of NO and PGI_2_ and significantly increased the release of ET-1. We also found that LSS significantly reduced the mRNA levels of eNOS and PGIS and significantly increased the ET-1 mRNA level. Intriguingly, DHP significantly improved these EC dysfunction phenomena induced by LSS. These results indicate that DHP may inhibit the occurrence of AS by improving LSS-induced EC dysfunction.

Mechanosensitive molecules play important roles in the induction of AS by LSS, such as HIF1*α*, VEGFR2, YAP1, and TWIST1. LSS activates these mechanically sensitive molecules, induces EC dysfunction, and promotes the development of AS. In the present study, we found that LSS significantly increased the mRNA levels of HIF1*α*, VEGFR2, YAP1, and TWIST1 and DHP significantly decreased the mRNA levels of HIF1*α*, VEGFR2, YAP1, and TWIST1. These results suggest that the effects of DHP in ameliorating EC dysfunction may be related to HIF1*α*, VEGFR2, YAP1, and TWIST1.

LSS can also cause oxidative stress and promote the occurrence of AS [25]. In the present study, we found that LSS significantly reduced the activity of SOD and GSH and significantly increased the levels of ROS, MDA, and GSSG, breaking the “oxidation-reduction” balance of EC. Intriguingly, DHP significantly improved these oxidative stress phenomena induced by LSS. These results indicate that DHP may inhibit the occurrence of AS by improving LSS-induced oxidative stress.

AS is a chronic inflammatory disease in which a variety of immune cells are involved, and neutrophils are closely related to the initiation of early chronic inflammation in AS. Abnormal expression of adhesion molecules (e.g., ICAM-1 and VCAM-1) in endothelial cells causes neutrophil recruitment and mediates migration of neutrophils to vascular inflammation sites, accelerating the development of AS vascular wall inflammation [34]. Recently, compelling evidence indicates that ICAM-1 is critical for the recruitment of neutrophils via binding to neutrophil *β*2 integrin at sites of the inflamed endothelium. *β*2 integrin LFA-1 (lymphocyte function-associated antigen-1, *α*_L_*β*_2_) and Mac-1 (macrophage-1 antigen, *α*_M_*β*_2_) are important molecules that mediate the recruitment of neutrophil in the inflammatory response. They have similar structures but have different physiological functions. LFA-1 was the prevailing ligand of ICAM-1 and mainly mediates the slow-rolling, stable adhesion and migration of neutrophil on vascular endothelial cells, whereas Mac-1 mainly mediates the crawling and polarization of neutrophil in blood vessels [35]. Furthermore, the activated *β*2 integrin-mediated neutrophil arrest and migration is a prerequisite for transcellular neutrophil. But they need binding to its cognate endothelial ligand ICAM-1 to mediated adhesion [36]. In the present study, we found that LSS significantly increased mRNA expression of the adhesion molecules of ICAM-1 and VCAM-1. And we also found that a large number of neutrophils were recruited in the low shear blood vessels of zebrafish (Figure 3). Importantly, DHP can significantly reverse this change. These results indicate that DHP may inhibit the occurrence of AS by improving LSS-induced inflammatory.

## 5. Conclusions

In summary, DHP exhibited the effects in LSS-induced EC dysfunction and HCD-induced hyperlipidemia, inflammation, and oxidative stress and inhibited plaque formation in AS. These results suggest that DHP may be a drug that can effectively prevent AS.

## Data Availability

All data used to support the findings of this study are included within the article.

## Abbreviations

Ang2: Angiopoietin 2
AS: Atherosclerosis
BMP4: Bone morphogenetic protein 4
DHP: *Dendrobium huoshanense* C. Z. Tang et S. J. Cheng polysaccharide
eNOS: Endothelia NO synthase
d.p.f.: Days postfertilization
EC: Endothelial cell
ET-1: Endothelin-1
GAPDH: Glyceraldehyde-3-phosphate dehydrogenase
GSSG: Glutathione disulfide
GSH: Glutathione
HCD: High cholesterol diet
HIF1*α*: Hypoxia-inducible factor 1*α*
HUVECs: Human umbilical vein endothelial cell
MDA: Malondialdehyde
NOTCH1: Columba livia notch 1
ICAM-1: Intercellular cell adhesion molecule-1
IL-8: Interleukin-8
KLF2: Krüppel-like factor 2
LFA-1: Lymphocyte function-associated antigen-1
LSS: Low shear stress
Mac-1: Macrophage-1 antigen
NO: Nitric oxide
PGI_2_: Prostaglandin I2
PGIS: Prostaglandin I2 synthase
ROS: Reactive oxygen species
SD: Standard deviation
SOD: Superoxide dismutase
TC: Total cholesterol
TG: Triglyceride
TWIST1: Twist-related protein 1
VCAM-1: Vascular cell adhesion molecule-1
VEGFR2: Vascular endothelial growth factor receptor 2
YAP: Yes-associated protein.

## Supplementary Materials

Supplementary Materials Fig. S1: the different regions of blood vessels in zebrafish. Low wall shear stress (LSS) incline to occur in the near-caudal wall region of blood vessels of zebrafish (Region 4). Table S1: primers used for the quantitative real-time polymerase chain reaction.
