# Supplementary material for: Protective Activities of Dendrobium huoshanense C. Z. Tang et S. J. Cheng Polysaccharide against High-Cholesterol Diet-Induced Atherosclerosis in Zebrafish
Source: Oxid Med Cell Longev. 2020 Jul 8;2020:8365056. doi: 10.1155/2020/8365056 (PMC7366212; doi:10.1155/2020/8365056)

Supplementary Material

Supplementary Figure and Table.

Figure. S1. The different regions of blood vessels in zebrafish. Low wall shear stress (LSS) incline to occur in the near-caudal wall region of blood vessels of zebrafish (Region 4).

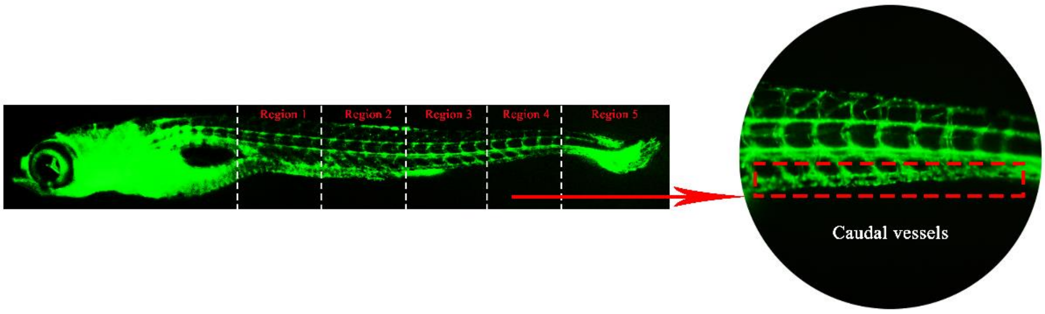

Table. S1. Primers used for the quantitative real-time polymerase chain reaction.

**Gene name** *Homo sapiens*	**Forward primer (5'->3')**	**Reverse primer (5'->3')**	**Gene bank**
Ang-2	AGTGGCTAATGAAGCTTGAGA	GTTTGCTCCGCTGTTTGGTT	NM_001118887.2
BMP4	ATCTTTGCTGTTGCTGGTGTC	CGTTATCCGAGGTGTTCGCT	XM_021477866.1
eNOS	AGCTGCCCTGATGGAGATGT	CCCGAACACACAGAACCTGAG	NM_000603.5
ET-1	TTGAGATCTGAGGAACCCGC	GCTCAGCGCCTAAGACTGTT	NM_001168319.2
HIF1α	CTGAGAGGTTGAGGGACGGA	GACGTTCAGAACTTATCCTACCATT	NM_001243084.1
ICAM-1	ATGGCAACGACTCCTTCTCG	GCCGGAAAGCTGTAGATGGT	NM_000201.3
KLF2	GAAGTACCCAGCCACAGCAT	ATGCGCTTCTCCAATCGGTA	NM_001042722.2
NOTCH1	CGTGGTGGACCGCAGAG	CTGGCACGATTTCCCTGACC	NM_017617.5
PGIS	CTGTGCTTGATAGCGTGCTG	GTCGCAGGTTGAATTCTCGC	NM_000961.4
TWIST1	CTTCTCGGTCTGGAGGATGG	GAAACAATGACATCTAGGTCTCCG	NM_000474.4
VCAM-1	GGTCGTGATCCTTGGAGCCT	CTTAGGAAAAGAGCCTGTGGTG	NM_001199834.1
VEGFR2	GGTTGTGTATGTCCCACCCC	TACCAGTGGATGTGATGCGG	NM_002253.3
YAP1	CCCTCGTTTTGCCATGAACC	GTTGCTGCTGGTTGGAGTTG	NM_001130145.3
GAPDH	TCGGAGTCAACGGATTTGGT	TTCCCGTTCTCAGCCTTGAC	NM_002046.7

Graphical Abstract

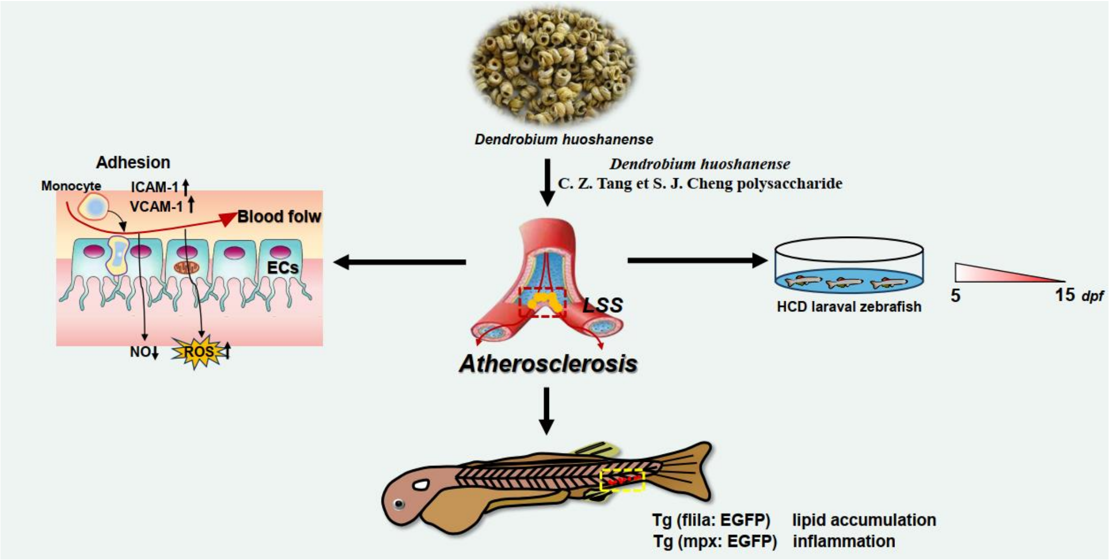

Supplement: Supplementary Materials — Fig. S1: the different regions of blood vessels in zebrafish. Low wall shear stress (LSS) incline to occur in the near-caudal wall region of blood vessels of zebrafish (Region 4). Table S1: primers used for the quantitative real-time polymerase chain reaction. [file 8365056.f1.pdf]
